# Unaided reading speed in pseudophakic patients after emmetropic monofocal intraocular lens implantation

**DOI:** 10.1038/s41433-025-04048-x

**Published:** 2025-10-23

**Authors:** Mayank A. Nanavaty, Ritika Mukhija, Zahra Ashena, Catey Bunce, David J. Spalton

**Affiliations:** 1 Sussex Eye Hospital, University Hospitals Sussex NHS Foundation Trust, Eastern Road, Brighton, UK; 2 Brighton & Sussex Medical School, University of Sussex, Falmer, Brighton, UK; 3 London School of Hygiene & Tropical Medicine, London, UK; 4 Kings College, London, UK

**Keywords:** Outcomes research, Diagnosis

## Abstract

**Aim:**

This study aimed to assess the reading speed and font size in pseudophakic eyes following implantation of a spherically neutral monofocal intraocular lens (IOL) aimed at an emmetropic refractive outcome.

**Methods:**

This was a prospective, single-eye study on patients undergoing routine cataract surgery with a spherically neutral monofocal IOL and expected to achieve 20/40 or better-unaided vision. Eyes with surgical complications or co-existing ocular pathology were excluded. At 3–9 months post-surgery, manifest refraction, uncorrected and best-corrected distance (UCDVA and BCDVA), and uncorrected near (UCNVA) LogMAR acuity, spherical equivalent (SEQ) were assessed. Uniocular unaided reading speed and smallest print size were assessed using the Salzburg Reading Desk (SRD) at 40 cm.

**Results:**

Median UCDVA, BCDVA, UCNVA, and SEQ of 301 patients (301 eyes) with age of 75 years (IQR:70 to 82) were 0.14 LogMAR (IQR = 0.02–0.24), 0 LogMAR (IQR = −0.08 to 0.02), and 0 D (IQR = −0.13 D to −0.07), respectively. The unaided reading speed was 100–150 wpm in 42.8% (129 patients), followed by 151–200 wpm in 26.1% (78 patients). The median unaided reading speed with SRD was 130 wpm (IQR: 101.50 to 165.50). 30.9% (93 patients) read 0.4 to 0.5 LogMAR, followed by 10.8% (33 patients) for 0.3 to 0.4 LogMAR and 7.4% (22 patients) for 0.2 to 0.3 LogMAR. The median smallest font size was 0.48 LogMAR (0.40 to 0.61 LogMAR).

**Conclusion:**

This study establishes the baseline data of reading speed and font size with monofocal IOL, which can be used by further studies on extended depth-of-focus or multifocal IOLs for comparison.

## Introduction

Cataract and refractive surgery have developed rapidly over the past decade [1]. Determining visual acuity is one of the most essential clinical examinations when dealing with the potential benefits of procedures to correct presbyopia [2–4]. Reading vision outcomes are usually measured with commonly available printed reading charts such as Jaeger charts [5]. There are relevant differences in print size between Jaeger charts of different manufacturers and even between charts of the same manufacturer [6]. Determining reading acuity is an essential clinical examination, mainly when discussing the potential benefits of presbyopia correction surgery [2, 7]. For patients who choose to undergo presbyopia correction surgery, uncorrected reading acuity and reading speed are the most important measures of success because the ability to read comfortably without any correction (i.e., spectacles or contact lenses) is their primary motivation for undergoing surgery.

Currently available near- and distance-vision tests meet the minimum requirements of international recommendations [8, 9], but standardising reading conditions with specific speed, between reading distance font size and lighting is not yet widely available. Representative of modern log-scaled reading charts, Radner was the first to implement sentence optotypes to minimise variations between the test items and to keep the geometric proportions as constant as possible at all reading distances [10]. The Salzburg Reading Desk (SRD) was introduced to further improve the assessment of reading tests by evaluating reading acuity at the best reading distance. With this device, a sentence will be taken into statistical account when the patient has read aloud, with a minimum reading speed of 80 words per minute (wpm), representing the lower limit for recreational, sense-capturing reading [11].

Intraocular lens (IOL) technologies have been advancing with newer enhanced monofocal, extended depth of focus, and trifocal lenses to address intermediate and near visual needs [12]. However, there seems to be a paucity of data on the reading speed of a conventional monofocal aspheric IOL [13, 14]. This study aimed to assess the reading speed and font size in pseudophakic eyes following implantation of a spherically neutral monofocal IOL aimed at an emmetropic refractive outcome.

## Patients and methods

Patients who underwent uneventful cataract surgery with a monofocal IOL with postoperative refraction aimed at emmetropia were sequentially recruited in this prospective, non-blinded, non-randomised, single-eye cohort study the Sussex Eye Hospital, University Hospitals Sussex NHS Foundation Trust, Brighton, United Kingdom, between September 2019 and May 2022. This study, which forms part of the European Society of Cataract & Refractive Surgeons (ESCRS) funded MEROV study, was approved by the National Ethics Committee and followed the tenets of the Declaration of Helsinki. The study was registered with www.Clinicaltrials.gov (ID: NCT04011696). Part 1 of the MEROV study describing the incidence of pseudoaccommodation and the factors responsible for this has already been published [15].

Inclusion criteria were uneventful cataract surgery with the postoperative potential to see 20/40 (0.3 LogMAR) unaided or better, no significant macular pathology and willingness to follow up at 3 months. This study relied on the visual outcome, and patients were recruited independently of the surgeon. Eligible patients coming to the cataract assessment clinics for their first or second eye were approached to participate in the study. The eye operated on first (out of the two) was enroled in the study. As this study aimed to assess the reading speed and font size of a monofocal IOL and not to assess the effects of binocular summation, only one eye of each patient was recruited. Exclusion criteria were patients under 20 years of age and any ocular co-morbidity, which would preclude an expected postoperative 20/40 unaided distance vision (e.g., amblyopia, corneal pathology including dry eyes, age-related macular degeneration, glaucoma, diabetic retinopathy, and previous ocular surgeries), anterior corneal astigmatism >2.5 D, posterior capsule opacification, surgical complications, inability to read English text or physical or mental inability to cooperate with the postoperative assessment.

### Recruitment and surgical procedure

Potentially eligible patients were given an invitation letter and information sheet when they arrived for surgery. Patients who agreed to participate were given a 3-month follow-up appointment in the research clinic. All participants had optical biometry with the IOLMaster® 500 (Carl Zeiss Meditec, Germany). Where biometry was not possible on IOLMaster® 500 due to the density of the cataract, A-scan ultrasound biometry (Accutome, Keeler, USA) was performed. A monofocal non-toric RayOne aspheric IOL (Rayner IOL, UK) was calculated using the SRK/T formula and an optimised A-constant of 118.8 was used in all cases. This is a single-piece preloaded acrylic hydrophilic IOL and is spherical aberration neutral. All surgeries were performed under topical anaesthesia as a day-case procedure. For patients with keratometric astigmatism of 0.75 D–2.5 D, peripheral corneal relaxing incisions (PCRIs) were performed using Donnenfield’s nomogram using a standardised and dedicated website, www.LRIcalculator.com, incorporating for the surgeon’s surgical induced astigmatism. A 2.85 mm superior or temporal incision (per surgeon’s protocol) was created, and the anterior chamber was filled with viscoelastic. The anterior capsulorhexis was created to overlap 360° of the IOL optic edge. Following successful phacoemulsification and bimanual irrigation & aspiration, the capsular bag was filled with viscoelastic, the IOL was implanted, and the anterior chamber was filled with a balanced salt solution. Patients were discharged on a tapering dose of G. Tobradex® (Alcon Laboratories, Fort Worth, Texas, USA) for one month.

### Follow-up assessments and outcome measures

Patients were followed up at 3 months after surgery. If this was delayed due to COVID-19-related restrictions, follow-ups were arranged up to 9 months post-surgery before the appearance of posterior capsule opacification. Uniocular uncorrected distance LogMAR (UCDVA) was recorded on the ETDRS LogMAR chart at 4 m at ambient room illumination. Manifest refraction and best-corrected distance vision (BCDVA) were assessed. An OCT scan was performed to establish macular integrity. Pupil size, keratometry, corneal topography and wavefront aberrometry were assessed on the iTrace aberrometer (Tracey Technologies, USA) using Software version 6.2.2.

Unaided (not even correcting the distance vision) near reading speed and the smallest readable print size for each patient were measured using a Salzburg Reading Desk (SRD) monocularly, which uses standardised measurements of reading performance with standardisation of illumination (100%), contrast (100%), and inclination. Tracking of the sound waves calculates the reading speed in words per minute (wpm). A stopwatch incorporated into the device records the reading duration, expressed in seconds. For this study, the reading distance was fixed at 40 cm and continuously monitored by a sticker on the patient’s forehead during the procedure using video stereophotogrammetry. The software calculated reading acuity in logMAR by considering the reading distance and the print size of the smallest readable sentence [16]. Functional reading was taken as the smallest font size read at 80 words per minute (wpm); the maximum reading speed font size was also measured. The iTrace® and SRD assessments were done in the same room for all patients in mesopic light conditions (3 cd/m^2^).

This study’s primary objective was to estimate the unaided reading speed in pseudophakic eyes with a monofocal lens. The secondary objectives were identifying the font size on SRD, residual sphere, cylinder/astigmatism, keratometric astigmatism, pupil size, total spherical aberration, and total vertical coma.

Sample size calculation is already described in the previous publication of this study [15]. All data were recorded on Microsoft Office Excel® (Microsoft® Corporation, USA). Means and standard deviations were used for quantitative data that were normally distributed (assessed by inspection of the histogram), and medians and interquartile ranges were used for non-normally distributed quantitative data. StatPlus:mac® (AnalystSoft Inc. Version v7) and Stata v 17.0 (Texas, USA) were used for all statistical analyses. A *p* value of <0.05 was considered statistically significant.

## Results

A total of 412 patients were recruited. Data were available on 301 patients. The median age was 75 (IQR: 70–82). Males were 45.2%, and 54.8% were females. Two patients died, and 109 withdrew or declined to follow up due to pandemic-related reasons. The reasons for withdrawal or decline in 109 patients were: too worried to attend hospital during the pandemic (35); developed new systemic morbidities (8); would like to be fully vaccinated before they attend (6); were shielding as they were living with vulnerable person (8); developed mobility issue due to new medical condition (4); had multiple surgeries recently (4); partner passed away due to COVID (6); could not wait for the entire set of research test to be completed on the day (2); uncontactable (15); appointment time clashed with other hospital appointments (3); fear of catching COVID during travel to the hospital (7); moved outside the county (6); and missing consent form in notes (5). There was no significant difference between the baseline characteristics of the 301 patients with the data available and 111 patients without data [15]. The majority of the patients (94.8%) in this study were Caucasians, representing the ethnicity of the local hospital patient population in Sussex, England [15].

The median unaided reading speed with SRD was 130 wpm (IQR: 101.50–165.50). Table 1 shows the frequency distribution of the unaided reading speed. Median reading speed was between 100 and 150 wpm in 42% of the patients (Table 1). Less than 35% of patients had a median reading speed above 150 wpm (Table 1).

**Table 1:** Uncorrected reading speed and the frequency of patients.

Uncorrected reading speed (WPM)	Percentage (%)	Cumulative percentage (%)
0–50	4	4
50–100	20	24
100–150	42	66
150–200	26	92
200–250	7	99
250–300	<1	99
300–350	<1	100

The secondary outcomes are described in Table 2. The frequency distribution of the smallest font size readable at the above reading speed is shown in Table 3. Therefore, the median uniocular reading speed in patients with aspherically neutral monofocal IOL is 130 wpm for the font size of 0.48 LogMAR.

**Table 2:** Secondary outcome measures.

	All eyes
	*N* = 301
	Median (IQR)
Age (years)	75 (70–82)
UCDVA (LogMAR)	0.14 (0.02–0.24)
BCDVA (LogMAR)	0.00 (−0.08 to 0.02)
Sphere (dioptres)	0.25 (0.00–0.75)
Cylinder (dioptres)	−0.75 (−1.25 to −0.50)
Axis of the cylinder (degrees)	90.00 (70.00–110.00)
Spherical equivalent (Dioptre)	0.00 (−0.38 to 0.38)
Total eye spherical aberration (µm)	0.02 (0.00–0.04)
Total eye vertical coma aberration (µm)	0.02 (0.01–0.05)
Mesopic pupil size (mm)	4.05 (3.60–4.65)
Smallest reading font size (LogMAR)	0.48 (0.40–0.61)

**Table 3:** Frequency distribution of reading font sizes.

Font size (LogMAR)	Percentage (%)	Cumulative percentage (%)
0–0.1	1.7	1.7
0.1–0.2	3.1	4.8
0.2–0.3	7.2	12.1
0.3–0.4	10.7	22.8
0.4–0.5	30.7	53.6
0.5–0.6	19.0	72.6
0.6–0.7	13.5	86.1
0.7–0.8	5.5	91.6
0.8–0.9	4.8	96.5
0.9–1	2.4	98.9
1–1.1	1.0	100

## Discussion

In today’s modern society, reading is an indispensable tool in everyday life. It has been shown that the loss of reading ability can reduce the quality of life [10, 17–19]. Analysis of reading performance can be focused on reading acuity and reading speed. Some studies measured reading speed using tests such as the Minnesota Low Vision Reading Test (MNRead) [17, 20] or the more recently developed Bailey-Lovie test, which allows the simultaneous measurement of reading speed and reading acuity [19]. In concordance with these tests, the Spanish Radner test [18] was developed to provide a standardised measurement of reading acuity and speed (uses a logarithmic scale, the logRAD scale, which is equivalent to the LogMAR). The SRD [7] was also developed for better use of Radner Reading Charts (University Eye Clinic, Paracelsus Medical University of Salzburg, Austria); it provides constant test lighting of 500 lux, control of the near reading distance, and automated calculation of the reading speed and acuity. The small letter size of the running text of newspapers or inserts in drug packages is normally between 1.5 mm and 3.0 mm (approximately equal to 0.4 LogMAR and 0.7 LogMAR at a standard viewing distance of 40 cm) [21]. Therefore, patients who can read print sizes of 1.5 mm minimum should be able to satisfactorily manage all types of everyday reading tasks under photopic conditions. It has been reported in various studies that loss of reading ability can have a significant impact on the patient’s quality of life [17, 22]. Hence, it is important that optimal reading ability is achieved after cataract surgery, which can be evaluated by measuring reading acuity and reading speeds, which are the aspects of one’s ability to read adequately. Using an SRD, our study found the median uncorrected reading speed of 130 wpm (IQR: 101.50–165.50) (Table 1) at a median font size of 0.48 LogMAR (IQR: 0.40–0.61 LogMAR) (Table 2) at a fixed distance of 40 cm in standard mesopic light condition. Our study recorded a median mesopic pupil size of 4.05 mm (IQR: 3.60–4.65 mm) (Table 2). How does this compare to the reading parameters in phakic presbyopic eyes and eyes with monofocal, multifocal, and extended depth of focus (EDOF) IOLs using SRD?

**Fig. 1:**
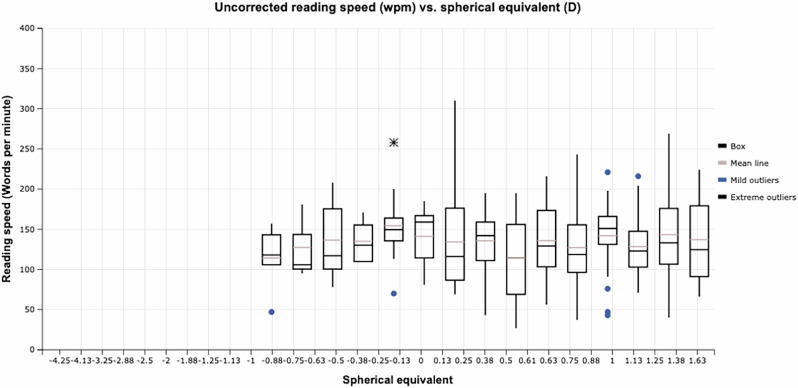
Box plot of uncorrected reading speed (Words per minute) versus postoperative spherical equivalent (dioptres). (Mild outliers: Values within the inner fence but within the outer fences, which are below Q1 - 1.5 × IQR but above Q1 - 3 × IQR and values above Q3 + 1.5 × IQR but below Q3 + 3 × IQR. Extreme outliers: Values falling beyond the outer fence, which are values below Q1 - 3 × IQR (lower extreme outliers) and above Q3 + 3 × IQR (upper extreme outliers. Q1 = first quarantile; Q3 = third quarantile and IQR = interquarantile range).

**Fig. 2:**
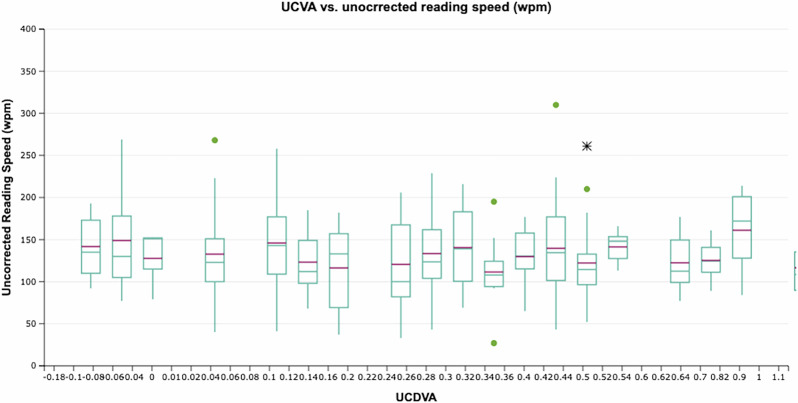
Box plot of uncorrected reading speed (Words per minute) versus uncorrected distance visual acuity (UCDVA). (Mild outliers: Values within the inner fence but within the outer fences, which are below Q1 - 1.5 × IQR but above Q1 - 3 × IQR and values above Q3 + 1.5 × IQR but below Q3 + 3 × IQR. Extreme outliers: Values falling beyond the outer fence, which are values below Q1 - 3 × IQR (lower extreme outliers) and above Q3 + 3 × IQR (upper extreme outliers. Q1 = first quarantile; Q3 = third quarantile and IQR = interquarantile range).

Conventionally, it was thought postoperative myopia was responsible for good reading speed. We plotted the relationship between uncorrected reading speed on SRD and postoperative myopia in Fig. 1, which shows no relationship. If postoperative myopia was the only factor determining their uncorrected reading speed on SRD, then the patients with better uncorrected reading speed should have worse UCDVA. We plotted this relationship between uncorrected reading speed and uncorrected distance vision in Fig. 2, showing no relationship between these. This shows that it is not just myopia which is responsible for good reading in pseudophakic eyes. Our previous study found that a combination of low myopic refraction, small pupil size, smaller pre-operative axial length, and very low total spherical aberration was responsible for pseudophakic pseudoaccommodation [15].

In phakic presbyopes (mean ± standard deviation; age: 45 ± 3.71 years (range: 38–49 years)) with distance correction only, Arad et al. [16] found the maximum reading speed of 178.48 ± 43.97 wpm (93–259 wpm) for the reading acuity of 0.41 ± 0.16 LogMAR (Range 0.03–0.71 LogMAR) at the reading distance of 44.4 ± 9.66 cm (26.7–63.1 cm). Although Arad et al. [16] found better reading speed than our study in pseudophakic eyes, the mean reading distance was not standardised and was further away than our study (i.e., more than 40 cm with SRD). In pseudophakic patients with monofocal IOLs, Rasp et al. [13] used AcriSmart 36 A IOL (now known as AT-Torbi, Carl Zeiss, Germany) and found a mean(SD) uncorrected reading speed of 148(40) wpm and the smallest print size of 0.47±0.15 LogMAR. Their reading speed was faster than ours (Table 1); however, their reading distance was 38.9 ± 8.4 cm in bright light without measuring pupil sizes. and shorter than ours (i.e., less than 40 cm) [13]. In our previous report on the MEROV study, we found that to achieve good unaided distance and reading in pseudophakic eyes, several factors work in combination, such as a low myopic spherical equivalent, lower total spherical aberration, shorter preoperative axial length, and smaller pupil [15]. However, in the MEROV study, eyes which had 20/40 unaided distance and 0.3 LogMAR for near 40 cm had a median reading speed of 106.00 wpm (range: 73.00–142.00 wpm) compared to the rest of the study sample, which had a median reading speed of 133.0 wpm (range: 104.25–167.00 wpm) with a larger median font size of 0.50 LogMAR (range: 0.45–0.69 LogMAR) [15].

In patients with pseudophakic multifocal IOLs, many studies have evaluated reading performance using the SRD [14, 23]. Rasp et al. [13] used trifocal AT LISA IOL (Carl Zeiss, Germany), bifocal ReStor (Alcon Laboratories, USA), bifocal ReZoom (Johnson & Johnson, USA) and Tecnis multifocal (Johnson & Johnson, USA) and found a mean uncorrected reading speed of 178 ± 50 wpm, 147 ± 35 wpm, 152 ± 40 wpm and 139 ± 32 wpm and the smallest print size of 0.23 ± 0.10 LogMAR, 0.29 ± 0.15 LogMAR, 0.40 ± 0.16 LogMAR and 0.27 ± 0.13 LogMAR at a reading distance of 31.6 ± 4.5 cm, 31.0 ± 5.6 cm, 37.1 ± 7.3 cm and 32.1 ± 5.4 cm, respectively. Jonker et al. [24] evaluated reading function by using the SRD for Finevision Micro F trifocal IOL (PhysIOL, Liege, Belgium) and the Acrysof Restor IQ + 3.0 dioptres bifocal IOL (Alcon Laboratories, USA). They found the reading speed of 170.8 ± 41.3 wpm and 154.0 ± 48.8 wpm with the smallest print size of 0.06 ± 0.10 LogMAR and 0.07 ± 0.07 LogMAR at the reading distance of 39.1 ± 5.39 cm and 36.4 ± 1.64 cm, respectively [24]. The reading speed of all the bifocal and trifocal lenses in the above studies is more than our finding in monofocal lenses, and the reading distance is shorter than 40 cm too (Table 2), which is attributable to the diffractive design of the IOLs.

Studies on patients with extended depth of focus (EDOF) IOLs look at reading with Radner’s reading test charts used in SRD. Sandoval et al. [25], using Tecnis Symfony IOL, compared binocular reading speed in two groups where the first group had EDOF IOLs implanted for distance emmetropia in both eyes, and the second group had micro-monovision with emmetropia in the dominant eye and −0.5 D in the non-dominant eye. They found the mean reading speed to be around 138 wpm and 142 wpm in emmetropia and micro-monovision groups, respectively, for the font size of 0.48 LogMAR (extrapolated from the figure) [25]. After bilateral implantation of Tecnis Symfony IOL implantation with mini monovision of −0.75 D, Ganesh et al. [26] found a mean binocular reading speed of 122.84 ± 33.50 wpm at 40 cm with 0.132 ± 0.13 LogMAR at 6 months. They also found that the reading speed improved from 1 to 6 months, and the font size decreased with time [26].

Comparing the above evidence in the literature with our study, it is evident that multifocal IOLs give good reading speed at smaller font sizes. However, there seems to be a very small difference between the uniocular median reading speed found in our study with monofocal IOLs and binocular reading speeds with EDOF IOLs in the literature, as shown above. Differences in the age range of the patients in various studies may affect the reading performances with various IOLs compared to our study. This should be taken into account when comparing our study results with others. This study is the first large sample study looking at the reading parameters on SRD in pseudophakic eyes with aspherically neutral monofocal IOLs. However, there are some limitations; namely, it only studies uniocular parameters. We already know that binocular reading parameters will be better than uniocular [27]; the study aimed to ascertain the baseline reading speed and font size for a monofocal pseudophakic eye. The other potential limitation is the fixed reading distance of 40 cm, which may not have given each patient their optimal reading distance. This was chosen as it is the standard reading distance where reading is assessed for all monofocal and premium IOLs [28]. Our postoperative follow-up measurements were between 3 and 9 months rather than 3 months due to the pandemic. Preoperative measurements of reading speed were not taken as the patients had an established diagnosis of cataracts. Furthermore, several factors may affect reading comprehension, such as working memory [29], cognitive health [30], thematic knowledge [29], and educational level [29], which were not assessed in this study. Further studies are needed to compare the uniocular reading speed after enhanced monofocal and EDOF IOLs and to compare it with standard monofocal IOLs.

In conclusion, this study establishes the baseline data of reading speed and font size with monofocal IOL, which can be used by further studies on extended-depth-of-focus or multifocal IOLs for comparison. This does not seem too far from the binocular reading speed with EDOF IOLs at the same font size.

## Summary

### What was known before

- The minimum reading speed for sense capturing and recreational purposes is 80 wpm.
- Binocular reading speed after bilateral extended depth of focus (EDOF) IOLs is between 132 and 138 wpm at 40 cm.

### What this paper adds

- The median reading speed with conventional monofocal IOL is 130 wpm with a font size of 0.48 LogMAR at 40 cm.
- Our study’s median uniocular reading speed is not too dissimilar to binocular reading speed after bilateral EDOF IOL implantation.

## Data Availability

Data available upon reasonable request.

## Conference presentations

Presentation at American Society of Cataract & Refractive Surgery 2023, San Diego, California and European Society of Cataract & Refractive Surgery meeting 2023 in Vienna.
